# Performance of High-Layer-Thickness Ti6Al4V Fabricated by Electron Beam Powder Bed Fusion under Different Accelerating Voltage Values

**DOI:** 10.3390/ma15051878

**Published:** 2022-03-03

**Authors:** Hongxin Li, Xiaoyu Liang, Yang Li, Feng Lin

**Affiliations:** 1 Department of Mechanical Engineering, Tsinghua University, Beijing 100084, China; lhx12kf@163.com (H.L.); xiaoyu_liang@tsinghua.edu.cn (X.L.); liyangthu8@163.com (Y.L.); 2 Key Laboratory for Advanced Materials Processing Technology, Ministry of Education of China, Beijing 100084, China; 3 Bio-Manufacturing and Rapid Forming Technology Key Laboratory of Beijing, Tsinghua University, Beijing 100084, China

**Keywords:** electron beam powder bed fusion, accelerating voltage, Ti6Al4V, high layer thickness, properties

## Abstract

The electron beam powder bed fusion (EB-PBF) process is typically carried out using a layer thickness between 50 and 100 μm with the accelerating voltage of 60 kV for the electron beam. This configuration ensures forming accuracy but limits building efficiency. The augmentation of the accelerating voltage enlarges the molten pool due to the rise in penetrability, suggesting that a higher layer thickness can be used. Therefore, the effects of layer thickness and accelerating voltage were investigated simultaneously in this study to explore the feasibility of efficiency improvement. Ti6Al4V was fabricated by EB-PBF using layer thicknesses of 200 and 300 μm. Two accelerating voltage values of 60 and 90 kV were used to study their effects under expanded layer thickness. The results reveal that dense parts with the ultimate tensile strength higher than 950 MPa and elongation higher than 9.5% could be fabricated even if the layer thickness reached 300 μm, resulting in a building rate of up to 30 mm^3^/s. The expansion of the layer thickness could decrease the minimum bulk energy density needed to fabricate dense parts and increase the α platelet thickness, which improved the energy efficiency. However, expanding layer thickness had a significant negative effect on surface roughness, but it could be improved by applying augmented accelerating voltage.

## 1. Introduction

Electron beam powder bed fusion (EB-PBF) is a kind of additive manufacturing technology which uses electron beams to selectively melt the powder layer by layer based on three-dimensional (3D) digital models and form 3D parts [1,2]. Compared with subtractive manufacturing technologies, parts with low thermal stress and arbitrary complex structures can be fabricated by EB-PBF due to the high powder-bed temperature and support effect provided by the sintered powder bed during the manufacturing process. The recycling of the sintered powder greatly increases the material utilization rate. In recent years, metal parts with high mechanical properties have been successfully fabricated by EB-PBF using different materials [3,4,5,6,7,8,9], which demonstrates promising prospects in aerospace, medicine and other fields.

As a powder bed fusion process, layer thickness plays a key role in EB-PBF. Generally, the typical layer thickness for the EB-PBF process varies between 50 and 100 μm [10,11]. This is larger than the typical layer thickness between 20 and 50 μm used in laser powder bed fusion (L-PBF) which uses a laser as the heat source to melt the metal powder. A smaller layer thickness results in higher dimensional accuracy and surface roughness, but a lower building rate, which increases the building cost and limits its ability to form large-scale parts [12].

Serval research studies have demonstrated that a layer thickness between 100 and 250 μm could be used for the L-PBF process to fabricate dense parts with qualified mechanical properties [13,14,15,16,17,18]. A building rate up to 12 mm^3^/s could be reached, which is about 10 times larger than the typical L-PBF process [16]. These research studies showed that, when a higher layer thickness was applied, higher line energy was needed, which amplified the keyhole and splashing effects and resulted in pore defects. However, these phenomena could be suppressed by adopting a higher beam diameter, by which a more stable molten pool could be obtained [19]. These findings indicate that expanding layer thickness is an effective way to improve the building rate.

However, to date, most of those studies are carried out by L-PBF and few studies about the effects of high layer thickness on EB-PBF are reported because of the equipment limitations. The ability to fabricate parts using high layer thickness by EB-PBF still lacks validation and the effect of high layer thickness on EB-PBF-manufactured parts is still unclear. Tang et al. [20] fabricated the Ti–45Al–7Nb–0.3W alloy with a layer thickness of 200–300 μm using a self-made EB-PBF machine and obtained a relative density up to 98.3%. Rännar et al. [21] manufactured 316L stainless steel with a layer thickness of 100–200 μm using the A2 ARCAM machine, where the density of the sample with 200 μm layer thickness was 3.2% lower than that of the sample with 100 μm layer thickness. These results indicate that lack of fusion is the primary defect for EB-PBF parts manufactured with high layer thickness.

For the EB-PBF process, the power (*P*) of the electron beam is determined by the product of the electron beam current (*I*) and accelerating voltage (*U*). The accelerating voltage determines the speed of the electrons and is fixed at 60 kV in most EB-PBF equipment. However, previous research studies showed that the molten pool depth increased significantly when the accelerating voltage was augmented, which was caused by the higher penetrability of a higher speed electron beam [22,23]. Hence, when considering reducing unfused defects, higher accelerating voltage helps fabricate parts using high layer thickness.

In this study, the possibility of using high layer thickness (up to 300 μm) in EB-PBF process and the possibility where the accelerating voltage could be used to improve the forming ability of high layer thickness were investigated. An EB-PBF machine with an accelerating voltage varying between 60 and 90 kV was developed. Ti6Al4V parts were built using both accelerating voltage values using layer thicknesses of 200 and 300 μm, respectively. The effects of the parameters on the density, surface roughness, microstructure and tensile properties of the as-built samples were studied.

## 2. Experimental Procedure

### 2.1. Materials

**Figure 1:**
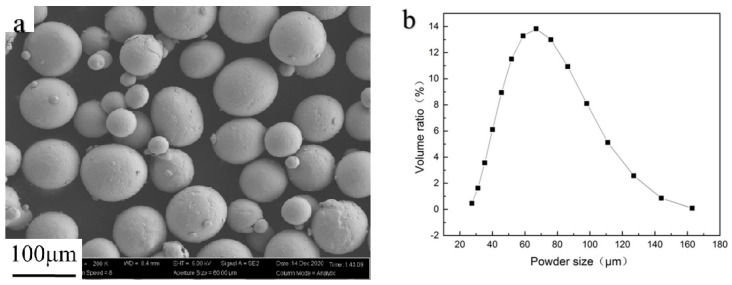
(**a**) Morphology of Ti6Al4V powder (reprinted with permission from Ref. [22], Copyright 2022 Elsevier); (**b**) powder size distribution of Ti6Al4V powder.

Gas-atomized Ti6Al4V Grade 23 alloy powder (Beijing AVIC Matt Co., Ltd., Beijing, China) with a size range of 45–105 μm was used for this study. The flowability was 25.9 s/50 g, which was measured using a hall flowmeter (BT-200; Bettersize Instruments Ltd., Dandong, China). The powder morphology is shown in Figure 1a; the powder size distribution was measured using a laser particle size analyzer (Mastersizer 3000, Malvern Panalytical Ltd., Worcester, UK) and is shown in Figure 1b; and the chemical composition is listed in Table 1.

### 2.2. Equipment and Manufacturing Process

The tests were carried out using EB-PBF-250 equipment developed by Tsinghua University [24,25]. The accelerating voltage of this equipment was variable between 60 and 90 kV. A Ti6Al4V alloy sheet with a thickness of 10 mm was used as the substrate. The substrate was preheated to 700 °C before the powder spreading process and the powder bed was preheated to 700 °C before the melting process. Building samples with a cross-section of 20 × 20 mm^2^ and a height of 8 mm were fabricated under a constant pressure of 0.1 Pa with argon backfilling.

**Figure 2:**
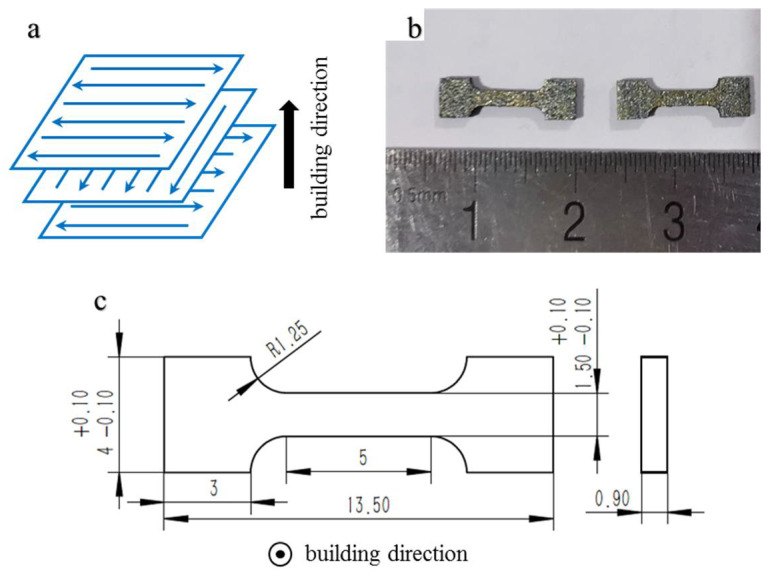
(**a**) Scanning strategy; (**b**) tensile samples; (**c**) dimensions (in mm) of tensile samples (reprinted with permission from Ref. [22], Copyright 2022 Elsevier).

An “S-mode” strategy was adopted for the melting process, as depicted in Figure 2a. The scanning direction between adjacent layers was rotated by 90° and the scanning direction between adjacent scanning lines was rotated by 180°. The bulk energy density *E* (J/mm^3^), referred to the energy obtained in unit volume by material during the electron beam melting, was used to characterize the heat input. It is calculated by

(1) $$E=\frac{UI}{hvL}$$

where *U* denotes the accelerating voltage (kV), *I* is the electron beam current (mA), *h* denotes the hatching space between adjacent scanning lines (mm), *v* denotes the scan speed (mm/s) and *L* is the layer thickness (μm). In this experiment, the scanning speed was fixed at 500 mm/s and the hatching space was 0.2 mm.

Two layer thicknesses, 200 and 300 μm, were used to investigate the effect of high layer thickness on the forming quality of the as-built samples. Besides the accelerating voltage of 60 kV, 90 kV was also used to evaluate the feasibility of forming specimens with high layer thickness using high accelerating voltage. In all experiments, the electron beam was focused to the smallest spot diameter that the equipment could achieve (approximately 400 μm at 60 kV and approximately 300 μm at 90 kV) during the melting process, which could suppress the keyhole and splashing effects because of the larger spot diameter than the spot diameter below 100 μm in L-PBF [19]. The processing parameters for all samples are listed in Table 2; three different bulk energy densities were used and the beam current was determined according to Equation (1).

### 2.3. Characterization

The sample densities were measured using the Archimedes principle with equipment ADVENTURER, AR423DCN. The nominal density of Ti6Al4V was calculated as 4.42 g/cm^3^ [26]. The surface roughness measurements were carried out using a 3D confocal microscope (Phase Shift MicroXAM-3D). The arithmetic average value (Ra) was used for the surface roughness analyses. During the surface roughness measurements, the measured direction for the lateral surface was parallel to the build direction and the measured direction for the upper surface was perpendicular to the scanning direction.

The surface morphologies and microstructures were observed using a Sigma 300 field emission scan electron microscope (FESEM), which operated at 5 kV. The samples were polished and then etched for 20 s with Kroll’s reagent (vol. 1% HNO_3_, vol. 2% HF, vol. 97% H_2_O) to better reveal the microstructure. Tensile samples, shown in Figure 2b, were tested using a Zwick Z2.5 TH tensile test machine at 25 °C. The strain rate was 0.5 × 10^−3^/s and the test direction was perpendicular to the building direction. The dimensions of the tensile samples are depicted in Figure 2c.

## 3. Results and Discussion

### 3.1. Relative Densities and Surface Morphologies

**Figure 3:**
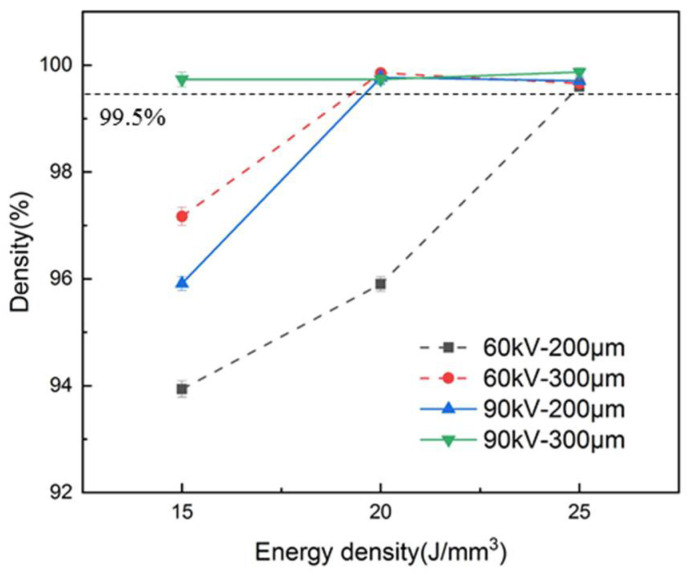
Relative densities of as-built samples.

The density results for the as-built samples are listed in Table 3 and plotted in Figure 3. The dense parts were defined with a density greater than 99.5 %. It can be observed that dense parts could be achieved at the bulk energy density of 25 J/mm^3^ for both layer thicknesses and accelerating voltages. Furthermore, when the layer thickness expanded from 200 μm to 300 μm, the minimum bulk energy density needed to fabricate dense parts reduced from 25 J/mm^3^ to 20 J/mm^3^ at the accelerating voltage of 60 kV and from 20 J/mm^3^ to 15 J/mm^3^ at the accelerating voltage of 90 kV.

**Figure 4:**
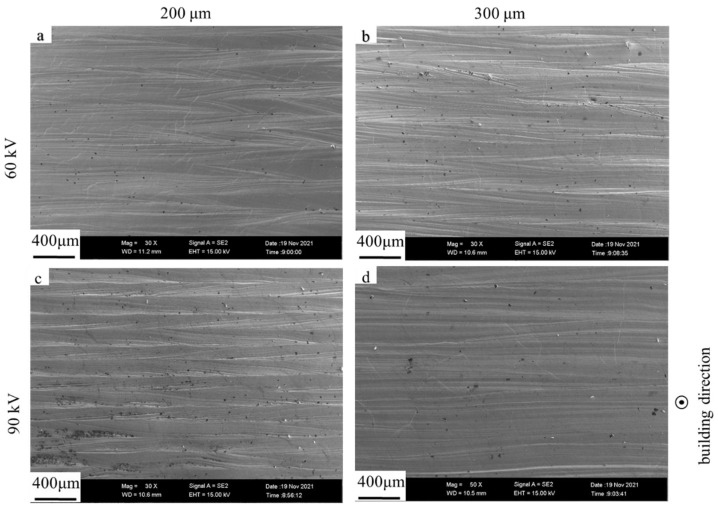
FESEM images of upper surface morphologies for different layer thicknesses and different accelerating voltage values with the bulk energy density of 25 J/mm^3^. (**a**) Sample 3; (**b**) sample 6; (**c**) sample 9; (**d**) sample 12.

**Figure 5:**
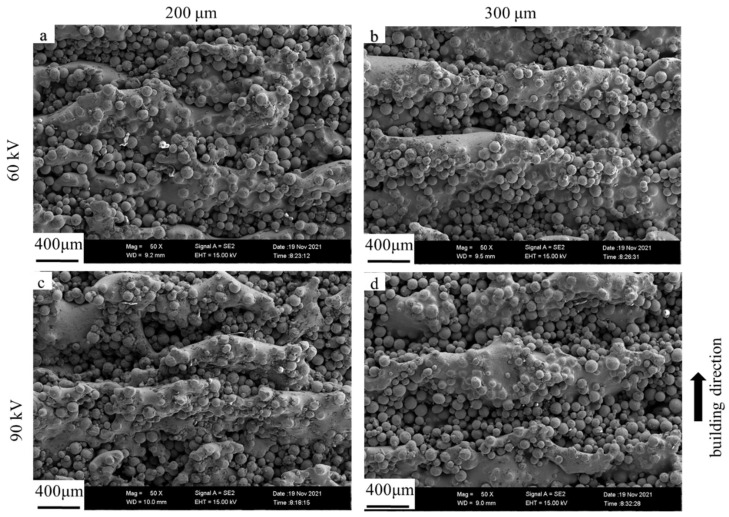
FESEM images of lateral surface morphologies for different layer thicknesses and different accelerating voltage values with the bulk energy density of 25 J/mm^3^. (**a**) Sample 3; (**b**) sample 6; (**c**) sample 9; (**d**) sample 12.

**Figure 6:**
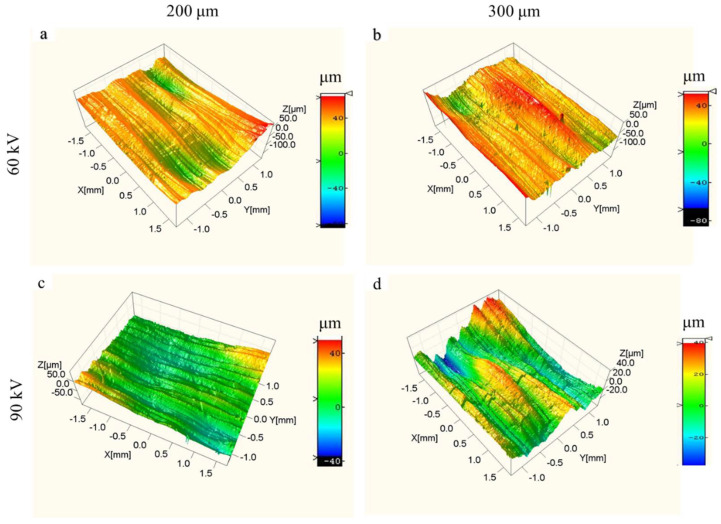
Three-dimensional morphologies of upper surface morphologies for different layer thicknesses and different accelerating voltage values with the bulk energy density of 25 J/mm^3^. (**a**) Sample 3; (**b**) sample 6; (**c**) sample 9; (**d**) sample 12.

**Figure 7:**
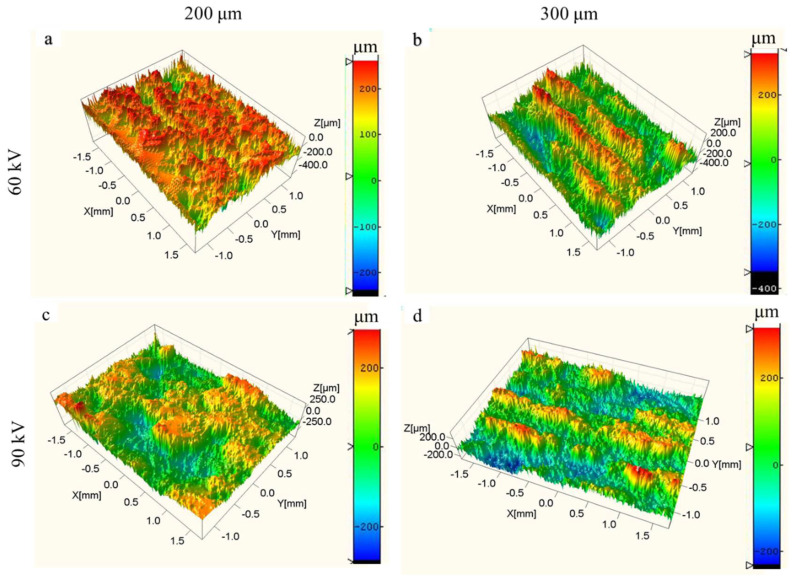
Three-dimensional morphologies of lateral surface morphologies for different layer thicknesses and different accelerating voltage values with the bulk energy density of 25 J/mm^3^. (**a**) Sample 3; (**b**) sample 6; (**c**) sample 9; (**d**) sample 12.

**Figure 8:**
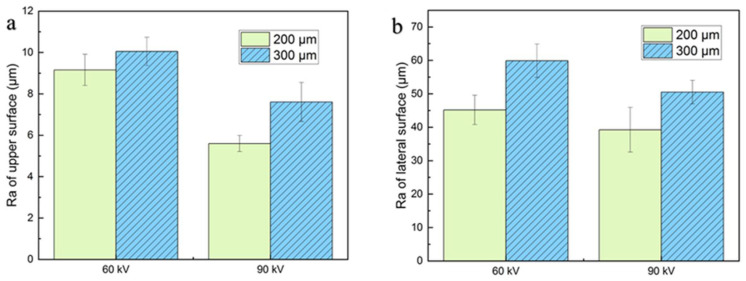
Surface roughness for different layer thicknesses and accelerating voltage values with the bulk energy density of 25 J/mm^3^: (**a**) upper surface; (**b**) lateral surface.

The upper and lateral surface morphologies of the dense samples fabricated with the bulk energy density of 25 J/mm^3^ under different layer thicknesses and different accelerating voltages were evaluated using FESEM (as shown in Figure 4 and Figure 5) and 3D confocal microscope (as shown in Figure 6 and Figure 7), respectively. The surface roughness values of those samples are plotted in Figure 8.

As shown in Figure 4 and Figure 6, the single-line scanning tracks could be observed, which caused the surface fluctuations and resulted in roughness (Ra) values between 5 and 10 μm of the upper surface. No significant differences could be observed for the four upper surfaces fabricated using different parameters. However, the surface roughness of the upper surface at higher layer thickness was slightly increased and decreased by augmenting the accelerating voltage from 60 to 90 kV, as shown in Figure 8a. Compared to the upper surface, the lateral surface was more uneven and had a greater surface roughness. The semi-elliptical cross-section of the molten pool, combining the step effect caused by the layer thickness, resulted in a structure with alternating peaks and valleys perpendicular to the building direction, as shown in Figure 5 and Figure 7. Furthermore, the fluctuation of the molten pool and the incompletely melted powder adhered to the lateral surface could also exaggerate the lateral surface roughness. These effects resulted in lateral surface roughness values between 39 and 60 μm. Moreover, the surface roughness of the lateral surface at higher layer thickness was also increased and decreased by augmenting the accelerating voltage from 60 to 90 kV, as shown in Figure 8b, which shows the same trend as the upper surface.

With the expansion of layer thickness, the stochastic effect of the powder bed and the step effect caused by the melting tracks intensify, which exaggerates the surface roughness. The augmentation of accelerating voltage enlarges the melting depth, which improves the stability of the melt pool and reduces the track fluctuation [27]. Therefore, augmenting the accelerating voltage improves the surface roughness.

**Figure 9:**
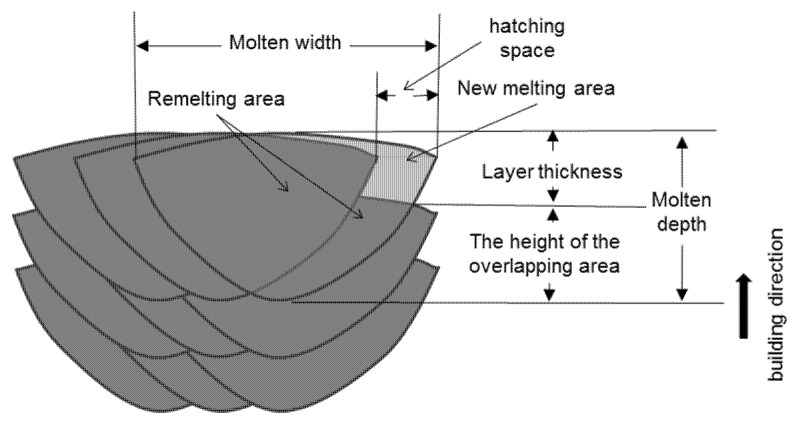
Schematic diagram of the melting tracks during the PBF process.

The reduction in the minimum bulk energy density required for dense part fabrication due to the escalation of layer thickness can be reflected by the upper surface morphologies. Figure 9 shows the schematic diagram of the melting tracks during the PBF process. The melting depth needed to achieve a dense part was much higher than the nominal layer thickness, which was caused by the stochastic effect that the effect layer thickness varies between zero and several times the nominal layer thickness [28]. Therefore, the section of the melting tracks could be divided into the remelting area and the new melting area and the remelting area should be significantly larger than the new melting area because the molten width and depth are significantly larger than the hatching spacing and layer thickness [22], as shown in Figure 9. When the layer thickness expanded from 200 μm to 300 μm, while the hatching spacing was maintained at 0.2 mm, the new melting area should have been extended by 50%. However, as shown in Figure 8a, the upper surface roughness (Ra) of the dense samples under different layer thicknesses showed a small difference, which indicates that dense parts could be obtained with only a small increase in the height of the overlapping area when the layer thickness expands and the increase in the remelting area should be less than 50%. These facts mean that, when the layer thickness is expanded by 50%, the increase in minimum line energy density (*P/v*) needed to achieve a dense part should be less than 50%, which results in a decrease in bulk energy density, as shown in Figure 3. In addition, the augmentation of the accelerating voltage enlarges the melting depth and results in a decrease in the bulk energy density to achieve dense parts [22].

### 3.2. Microstructure

**Figure 10:**
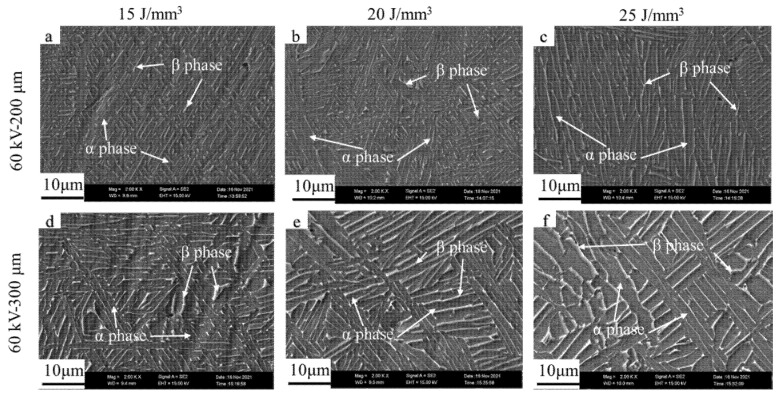
Microstructure of samples for accelerating voltage of 60 kV. From (**a**–**f**): sample 1 to 6.

**Figure 11:**
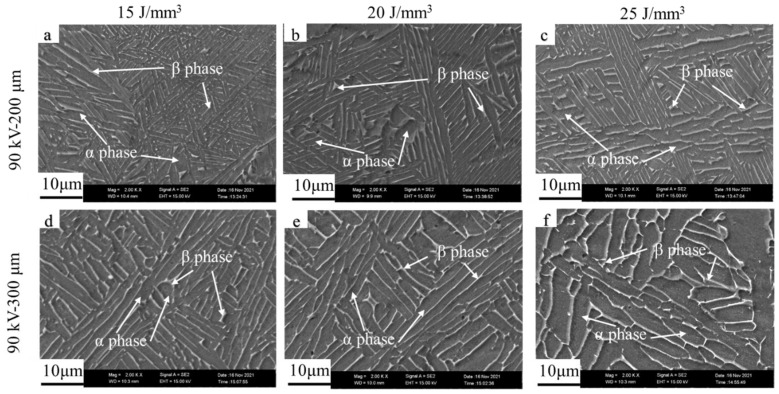
Microstructure of samples for accelerating voltage of 90 kV. From (**a**–**f**): sample 7 to 12.

Figure 10 and Figure 11 demonstrate the microstructures of the as-built samples for the accelerating voltage values of 60 and 90 kV at different layer thicknesses and bulk energy densities, respectively. It can be seen that the microstructure of all the as-built samples consisted of the *α* and *β* phases with a Widmanstätten-like structure; the gray *α* phase was surrounded by the white *β* phase, which showed no differences in phase compared with the microstructure obtained by low layer thickness [10].

**Figure 12:**
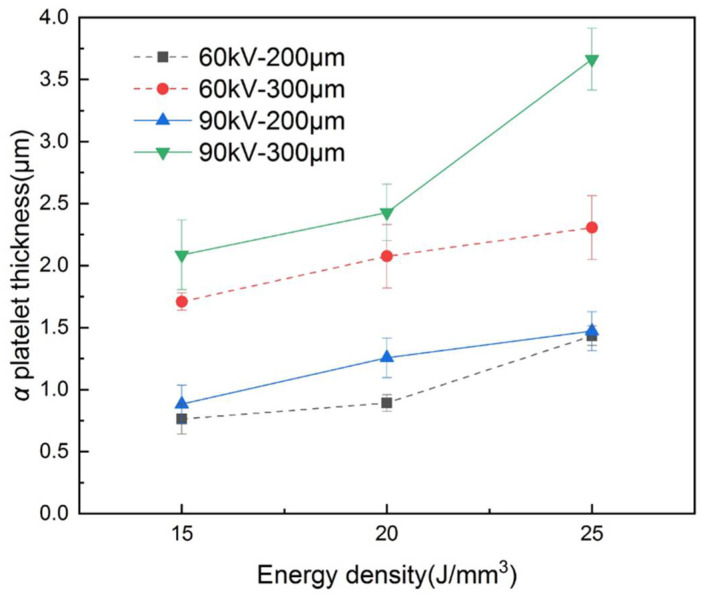
α platelet thickness of as-built samples under different parameters.

The difference in the microstructure of the as-built samples could be characterized by the change in the *α* platelet thickness, which is plotted in Figure 12. The *α* platelet thickness is determined by the cooling rate. When the cooling rate decreases, the *α* platelet has more time to grow up and coarsen [29]. Therefore, with the increase in the bulk energy density or the expansion of the layer thickness under the same bulk energy density, the line energy density (*UI/v,* defined as the beam power divided by scanning speed) increases and a larger molten pool can be obtained, which results in a lower cooling rate and a thicker *α* platelet, as shown in Figure 12. In addition, the *α* platelet thickness also increases with the augmentation of the accelerating voltage, which is caused by the higher energy efficiency and penetrability of the higher accelerating voltage [22].

### 3.3. Tensile Properties

**Figure 13:**
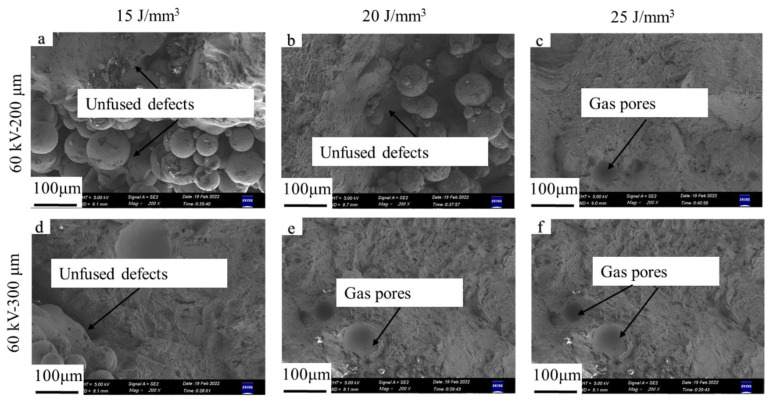
Fracture surfaces of tensile samples for accelerating voltage of 60 kV. From (**a**–**f**): sample 1 to 6.

**Figure 14:**
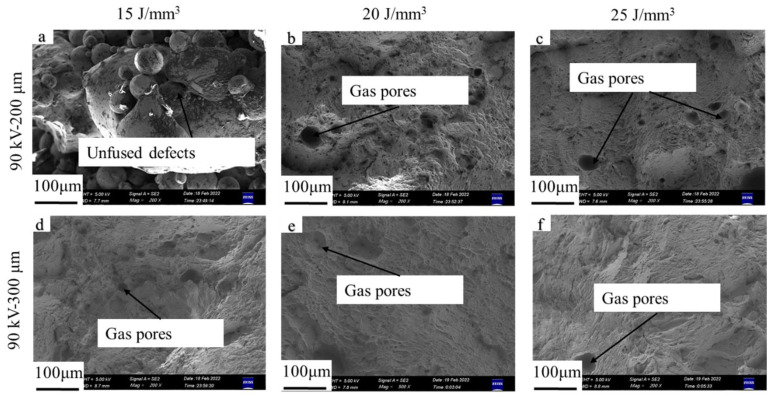
Fracture surfaces of tensile samples for accelerating voltage of 90 kV. From (**a**–**f**): sample 7 to 12.

The mechanical properties of the as-built samples are listed in Table 4. When the relative density was higher than 99.5%, an ultimate tensile strength higher than 950 MPa and elongation higher than 9.5% could be achieved, which agrees with the results obtained by EB-PBF process using layer thickness between 50 and 100 μm [11,22]. Porosity is the dominant factor in tensile properties [11]. The variation in tensile properties can be explained by unfused defects and entrapped gas in powder particles, as shown in Figure 13 and Figure 14. There were no significant differences between the layer thicknesses of 200 and 300 μm when the relative density was higher than 99.5%, which indicates that Ti6Al4V parts with desirable mechanical properties could be obtained with high layer thickness up to 300 μm via the EB-PBF process.

### 3.4. Building Rates

For the PBF process, the building rate is a key parameter that determines the forming efficiency and cost. The building rate can be defined as the volume melted per unit time, which can be calculated by

(2) B=hvL

where *B* denotes the building rate (mm^3^/s).

The typical building rates of Ti6Al4V for different PBF processes are listed in Table 5. For the L-PBF process, the building rate is lower than 5 mm^3^/s and can be raised up to 7.2 mm^3^/s by expanding the layer thickness from 30 μm to 200 μm [14]. As for the EB-PBF process, the typical building rate achieved by the ARCAM machine ranges from 2.5 to 30 mm^3^/s due to different scanning speeds [3]. In this research study, a building rate up to 30 mm^3^/s was achieved using the layer thickness of 300 μm and the scanning speed of 500 mm/s, which is 12 times the typical EB-PBF building rate at the scanning speed of 500 mm/s and equivalent to the building rate at the scanning speed of 6000 mm/s. It should be noted that, when considering the time spent on powder spreading and powder preheating process to build parts of the same height with a high layer thickness, it is significantly lower than that obtained using a low layer thickness; the forming efficiency using layer thickness of 300 μm and scanning speed of 500 mm/s should be greater than that obtained using layer thickness of 50 μm and scanning speed of 6000 mm/s.

## 4. Conclusions

In this research study, layer thicknesses up to 300 μm and accelerating voltage values up to 90 kV were used to fabricated Ti6Al4V samples by EB-PBF with different bulk energy densities. The relative densities, surface morphologies, microstructure and mechanical properties of the as-built samples were analyzed and compared using the layer thicknesses of 200 and 300 μm and the accelerating voltage values of 60 and 90 kV. The conclusions are as follows:

(1) Parts with relative density greater than 99.5%, ultimate tensile strength higher than 950 MPa and elongation higher than 9.5% were fabricated when the layer thickness expanded to 300 μm under both accelerating voltage values. The variation in tensile properties was mainly caused by porosities.

(2) With the increase in the layer thickness or accelerating voltage, the minimum bulk energy density needed to fabricate dense parts decreased due to the decrease in the proportion of the remelted area in the molten pool and the *α* platelet thickness increased due to the increase in the molten pool.

(3) A higher accelerating voltage enlarged the melting depth, which improved the surface finish even if it was impaired due to the expansion of layer thickness.

(4) A building rate up to 30 mm^3^/s without sacrificing the mechanical properties could be achieved when the layer thickness was 300 μm, the hatch space was 200 μm and the scanning speed was 500 mm/s.

## Figures and Tables

**Table 1:** Composition of Ti6Al4V powder.

Element	Ti	Al	V	Fe	O	N
wt.%	Balance	6.16	4.30	0.16	0.09	<0.01

**Table 2:** Process parameters of as-built samples.

Sample Number	Layer Thickness (μm)	Accelerating Voltage (kV)	Beam Current (mA)	Energy Density (J/mm^3^)
1	200	60	5	15
2	200	60	6.67	20
3	200	60	8.33	25
4	300	60	7.5	15
5	300	60	10	20
6	300	60	12.5	25
7	200	90	3.33	15
8	200	90	4.44	20
9	200	90	5.56	25
10	300	90	5	15
11	300	90	6.67	20
12	300	90	8.33	25

**Table 3:** Relative densities for all samples.

Sample Number	1	2	3	4	5	6
Relative density (%)	93.94 ± 0.15	95.90 ± 0.13	99.60 ± 0.10	97.17 ± 0.17	99.86 ± 0.04	99.66 ± 0.07
**Sample Number**	**7**	**8**	**9**	**10**	**11**	**12**
Relative density (%)	95.91 ± 0.10	99.77 ± 0.12	99.71 ± 0.05	99.73 ± 0.13	99.87 ± 0.08	99.88 ± 0.07

**Table 4:** Mechanical properties for as-built samples.

Sample Number	Layer Thickness (μm)	Accelerating Voltage (kV)	Energy Density (J/mm^3^)	Yield Strength (MPa)	Ultimate Tensile Strength (MPa)	Elongation (%)
1	200	60	15	277 ± 21	283 ± 17	1.0 ± 0.1
2	200	60	20	582 ± 11	590 ± 13	0.5 ± 0.1
3	200	60	25	969 ± 7	1077 ± 3	9.2 ± 0.4
4	300	60	15	519 ± 114	590 ± 115	1.6 ± 0.2
5	300	60	20	962 ± 2	1072 ± 5	9.3 ± 1.9
6	300	60	25	961 ± 26	1082 ± 29	10.3 ± 0.6
7	200	90	15	285 ± 19	310 ± 37	2.9 ± 0.2
8	200	90	20	839 ± 6	950 ± 7	13.4 ± 1.1
9	200	90	25	840 ± 20	962 ± 8	10.4 ± 0.3
10	300	90	15	927 ± 15	1034 ± 28	9.9 ± 1.9
11	300	90	20	877 ± 13	994 ± 5	11.3 ± 0.2
12	300	90	25	943 ± 11	1054 ± 3	10.1 ± 0.6

**Table 5:** Building rate of different technologies.

Layer Thickness (μm)	Scanning Speed (mm/s)	Hatch Space (mm)	Building Rate (mm^3^/s)	Manufacturing Technology	Reference
30	500	0.2	3	L-PBF	[30]
200	60	0.6	7.2	L-PBF	[14]
50	500–6000	0.1	2.5–30	EB-PBF	[3]
200	500	0.2	20	EB-PBF	In this research
300	500	0.2	30	EB-PBF	In this research
